# Lifelong learning and nurses’ continuing professional development, a metasynthesis of the literature

**DOI:** 10.1186/s12912-021-00579-2

**Published:** 2021-04-14

**Authors:** Mandlenkosi Mlambo, Charlotte Silén, Cormac McGrath

**Affiliations:** 1 Jersey General Hospital, St Helier, Jersey; 2 Department of LIME, Karolinska Institutet, Stockholm, Sweden; 3 Department of Education, Stockholm University, Stockholm, Sweden

## Abstract

**Background:**

Continuing professional development (CPD) is central to nurses’ lifelong learning and constitutes a vital aspect for keeping nurses’ knowledge and skills up-to-date. While we know about the need for nurses’ continuing professional development, less is known about how nurses experience and perceive continuing professional development. A metasynthesis of how nurses experience and view continuing professional development may provide a basis for planning future continuing professional development interventions more effectively and take advantage of examples from different contexts. The aim of this paper is to conduct such a metasynthesis, investigating the qualitative research on nurses’ experiences of continuing professional development.

**Methods:**

A metasynthesis of the qualitative literature was conducted. A total of 25 articles fulfilled the inclusion criteria and were reviewed.

**Results:**

We determined five overarching themes, Organisational culture shapes the conditions, Supportive environment as a prerequisite, Attitudes and motivation reflect nurse’s professional values, Nurses’ perceptions of barriers and Perceived impact on practice as a core value. This metasynthesis highlights that nurses value continuing professional development and believe that it is fundamental to professionalism and lifelong learning. Moreover CPD is identified as important in improving patient care standards.

**Conclusions:**

Based on the metasynthesis, we argue that access to continuing professional development could be made more attainable, realistic and relevant. Expediently, organizations should adequately fund and make continuing professional development accessible. In turn, nurses should continue to actively engage in continuing professional development to maintain high standards of nursing care through competent practice. This paper highlights the perceived benefits and challenges of continuing professional development that nurses face and offers advice and understanding in relation to continuing professional development. We believe that this metasynthesis contributes with insights and suggestions that would be valuable for nurses and policy makers and others who are involved in nurse education and continuing professional development.

## Introduction

Health care professionals need to update their skills regularly and continuing education, or continued professional development (CPD) enables the renewal and updating of skills in health care settings. While we know about the need for CPD, less is known about how nurses experience and perceive CPD, and currently, there is no comprehensive global picture of how nurses view and experience CPD. A metasynthesis of the qualitative literature on nurses’ experiences of CPD may provide a basis for planning future CPD interventions more effectively and take advantage of examples from different contexts. This paper is organised in the following way; first we present the notion of CPD, we then use the United Kingdom, (UK) as a setting to offer an overview of the different mechanisms that exist in one specific health care setting, which may impact engagement with CPD. We acknowledge that similar mechanisms may exist in other health care settings and countries too, and identify the UK context, merely as a way to frame the paper. Subsequently, we conduct a metasynthesis of the qualitative literature addressing the topic of how CPD is experienced by nurses.

### Continued professional development

This section aims to unpack the notion of CPD, which exists in different forms and is driven, in part, by top-down requirements, but also, bottom-up, from the needs of practitioners. Continuing professional development (CPD) programmes are central to nurses’ lifelong learning and are a vital aspect for keeping nurses’ knowledge and skills up-to-date. The requirement for nurses to participate in CPD differs between European countries and elsewhere in the world and can be mandatory or voluntary [1, 2]. For example, CPD is mandatory in the U. K, Belgium, Spain, Australia and in some states in the United States of America, [2–4]. In these countries, nurses engage in CPD because it is a mandatory condition by nurse regulators for remaining registered to practice. However, in Sweden, Netherlands and Ireland nurses participate in CPD of their own volition [1, 3–5]. Table 1 provides an overview of some of the European countries which provide mandatory and non-mandatory CPD.

**Table 1:** Examples of mandatory and non-mandatory CPD in nursing in Europe (EFN, 2012)

Mandatory CPD Countries	Non-mandatory CPD Countries
Belgium	Denmark
Cyprus	Finland
Czech Republic	Germany
France	Greece
Italy	Ireland
Latvia	Netherlands
Lithuania	Norway
Romania	Poland
Slovakia	Portugal
Spain	Sweden
United Kingdom	

In jurisdictions where CPD is mandatory, nurses engage in continuing education by participating in professional development that is relevant to their areas of practice. Mandatory CPD, refers to “… the process of ongoing education and development of healthcare professionals, from initial qualifying education and for the duration of professional life, in order to maintain competence to practice and increase professional proficiency and expertise” ([6], p.1). CPD can sometimes refer to a learning framework and activities of professional development which contribute to the continual professional effectiveness and competence [7]. Broadly, CPD is related to continuing education, and continual learning, both formal and informal, which results in the acquisition of knowledge and skills transfer by the practising nurse with the aim of maintaining licensure and competent practice [8]. Learners can utilise a mixed style approach to learning depending on the circumstances and context of the learning environment [9–11]. To succeed in providing comprehensive care for their patients, nurses need to utilise the best evidence available to them [12–14]. This requires different modes of learning and ways of knowledge acquisition and construction. To achieve this, nurses can engage in different approaches of acquiring knowledge through CPD, through formal learning, courses or workshops as well as workplace informal learning, through self-reflection, appraising literature for best evidence through journal clubs and giving feedback to each other [5, 7, 15]. Informal learning is often volitional and is largely initiated and controlled by individual nurses with the intention to develop their knowledge and skills [16–18]. Due to its unstructured and, at times, unintentional manner, such learning is often acquired during interactions with colleagues and patients [19]. One of the advantages of on-site learning, both formal and informal is that learners can utilise expertise which are already available on the ward [5, 15]. On-site learning occurs often at the discretion and the willingness of managers to facilitate by providing time and space for learning to occur within the clinical areas. Even so, the fact remains that informal on-site learning is not an event but a continuous process, which draws from daily professional experiences. Lack of CPD trained nurses and ward needs, coupled with poor staffing levels, are cited as main barriers to informal workplace learning [5, 15]. Evidence from CPD literature indicates that many nurses prefer informal work-based methods of learning, noting that most meaningful learning occurs through interactions with their colleagues [20]. From a study by Clarke [21], it was noted that nurses found informal learning methods such as supervision, attending team meetings/briefings, mentoring and observations to be important. Ultimately, whichever delivery method is used for CPD, continuous professional development extends the practitioner’s professional ability beyond pre-registration training, qualification and induction, thereby potentially enhancing the practitioner’s practice.

### Continued professional development: the UK example

This next section aims to illustrate the different mechanisms that arise in one specific health care setting when implementing CPD on a national scale. We recognise that other mechanisms will exist in other contexts, and in places where CPD is not a formal requirement.

Today, nurses in the U.K. are required to engage in continuous learning in order to maintain competence as a means of keeping their licensure with their professional body, the Nursing & Midwifery Council (NMC) [22]. Since the 1980s, UK nurses and other allied health care professionals such as physiotherapists and occupational therapists have been required to engage in continuous professional development [23]. A justification for CPD has been the need to maintain professional registration to practice. For registered nurses in the UK, the requirement to engage in CPD came to the fore of continuing education in 1995. It was introduced by the then licensing body, the United Kingdom Central Council for Nursing, Midwifery and Health Visiting (UKCC) as post registration education and practice (PREP) [24]. Further to that, the Agenda for Change Reforms in 2003 introduced a system for linking pay and career progression to competency called the National Health Service Knowledge and Skills Framework [25]. The framework is linked to the individual nurse’s ability to demonstrate that they possess the necessary knowledge and skills to get promoted and be remunerated accordingly [25]. In the UK, further reforms to CPD were introduced in 2012 through the introduction of the Health Education England (HEE) in England [27]. Its mandate was to equip the NHS (National Health Service) workforce, including nurses with appropriate knowledge and skills to deliver high standard care to patients. The HEE’s role was to support workforce development by providing funding largely for nurses’ CPD. In 2016, PREP was replaced with revalidation, which still requires nurses to attend 35 h of CPD every 3 years [24, 26]. Revalidation is the process through with nurses and midwives continue as registrants with the Nursing and Midwifery Council (NMC) [25]. However, comprehensive HEE budget cuts have had a negative effect on nurse CPD initiatives [27]. CPD funding in UK was cut from 205 million pounds in 2015–16 to 83 million in 2017–18 [28, 29]. Consequently, nurses have struggled to fulfil revalidation requirements due to some authorities freezing access and refusing to give nurses time to attend CPD activities [27].

This previous section offers an insight into different push-pull mechanisms, in the UK alone. Statutory requirements are underpinned by the need for nurses to maintain and develop the knowledge and skills to meet the expected competence standards of practice in response to expanding nursing roles and global trends. Our experience suggests that local governing bodies may enforce similar measures in contexts where CPD measure are not formalised. Nurses may find themselves caught between a patchwork of statutory requirements and a need to develop their skills and knowledge. Consequently, while we know about the need for nurses’ continuing professional development, less is known about how nurses experience and perceive continuing professional development. Therefore we propose that a metasynthesis of the qualitative literature could be a part of forming such a comprehensive view and use the following three questions to examine the literature What is the reported value of CPD for nurses’ lifelong learning and its impact on nursing knowledge?, What are the conditions necessary for CPD?, and, What are the challenges faced by nurses when engaging in CPD?

## Method

In this study, a metasynthesis was used to investigate the qualitative literature [30, 31]. Metasynthesis is a form of systematic review method used to review qualitative studies in order to develop theory, to explore and understand phenomena or generate new knowledge, thereby creating meaning from that knowledge [32–36]. In this review, we present a metasynthesis based on the interpretation of qualitative results from topically related qualitative reports. In doing so we strive towards theoretical development, which according to Zimmer refers to the synthesis of findings into a product that is ‘thickly descriptive, and comprehensive’ and thus more complete than any of the constituent studies alone ( [30] p.313).

The results from metasynthesis studies may be used to underpin and inform healthcare policy, nursing practice and patient care. Furthermore, such information can be utilised by health care professionals involved in nursing education to inform planning and designing of training and educational programs. A number of steps are taken when conducting a metasynthesis [36] and involve;

a) bringing together a multidisciplinary team, in our case the team of three people includes two skilled medical education professional researchers with extensive experience in qualitative studies, including systematic reviews, moreover these two authors have more than 40 years of comprehensive experience of CPD in health care settings, two of the team are registered nurses and afford the team key insights into the context of nursing CPD, the team is spread across three institutions in two countries, finally, the team consisted of a search engine expert,

b) defining inclusive but manageable research questions, see the questions above;

c) conducting the systematic search, in our case this was conducted by the search engine expert, see Table 2 for the search criteria,

**Table 2:** Inclusion and exclusion criteria for the review

	Inclusion Criteria
Types of studies	Studies published between 2010 and 2019.
	Studies published in English language.
	Original studies using qualitative methods (describing themes raised by participants) i.e. seeking to understand nurses’ experience of CPD, describing the challenges and benefits of CPD from the nurse’s perspective, nurse lifelong learning
Types of participants	Nurses, nurse managers Nurse educators
	Nurses working in different settings and contexts Nurses in educational roles Nurse managers
Abbreviated Key words	Education, Nursing, Continuing Education, Continued Professional Development, Learning, lifelong learning, nurse*, qualitative research, interview as topic, focus groups, Narration, ethnograph* qualitative or questionnaire*, survey*
Example from Search	Field labels • exp./ = exploded controlled term • / = non exploded controlled term • .ti,ab,id. = title, abstract and author keywords • adjx = adjacent within x words, regardless of order * = truncation of word for alternate endingsMAINSUBJECT.EXACT(“Professional Continuing Education”) OR (MAINSUBJECT.EXACT(“Lifelong Learning”) OR MAINSUBJECT.EXACT.EXPLODE(“Staff Development”)) OR ti(((continuing or continued or inservice or professional) NEAR/2 (education or development or program* or training))) OR ti((learning NEAR/2 (lifelong or life-long or ongoing or on-going or self-direct* or self-motivat* or voluntary* or work-based)).) AND (MAINSUBJECT.EXACT.EXPLODE(“Nurses”) OR ti((nurse* NEAR/6 ((continuing OR continued OR inservice OR professional) NEAR/2 (education OR development OR program* OR training)))) OR ab((nurse* NEAR/6 ((continuing OR continued OR inservice OR professional) NEAR/2 (education OR development OR program* OR training)))) OR ti((nurse* NEAR/6 (learning NEAR/2 (lifelong OR life-long OR ongoing OR on-going OR self-direct* OR self-motivat* OR voluntary* OR work-based)))) OR ab((nurse* NEAR/6 (learning NEAR/2 (lifelong OR life-long OR ongoing OR on-going OR self-direct* OR self-motivat* OR voluntary* OR work-based))))) AND (MAINSUBJECT.EXACT(“Qualitative Research”) OR MAINSUBJECT.EXACT.EXPLODE(“Interviews”) OR MAINSUBJECT.EXACT(“Focus Groups”) OR MAINSUBJECT.EXACT(“Narration”) OR ti((audiorecording* OR ethnograph* OR fieldwork OR “field work” OR “focus group*” OR interview* OR “key informant*” OR narration* OR narrative* OR observation* OR qualitative OR questionnaire* OR survey*)) OR ab((audiorecording* OR ethnograph* OR fieldwork OR “field work” OR “focus group*” OR interview* OR “key informant*” OR narration* OR narrative* OR observation* OR qualitative OR questionnaire* OR survey*))) Narrowed by:decade: 2010–2019;Source type: Scholarly Journals;Language: English;Peer reviewed: Peer reviewed
	**Exclusion Criteria**
Types of studies	Articles published before 2010 Non-English language Studies using quantitative methodologies Systematic reviews Grey literature and conference papers Non-peer reviewed journal articles Theses or dissertations Editorials, commentary or opinion articles Articles testing tools for CPD Articles focused on career development or revalidation not CPD Non-nurse/ Other healthcare professionals CPD

d) quality assessment of the studies, this was done using the CASP (Critical Appraisal Skills Programme) criteria, weighting three levels (not met, partially met, totally met) where assessment was done by all three authors see Table 4, e) extracting data from the studies, see Table 3,

**Table 3:** Summary of articles with location cohort data collection method

Article	Location	n	Method/data collection	Respondents
Averlid, G. (2017). Norwegian nurse anesthetist perceptions of professional development and the influence of production pressure. AANA journal, 85 (5), 345.	Norway	14	Interviews	Nurses
Balls, P. (2010). What are the factors that affect band 5 nurses’ career development and progression? Nursing Times, 106 (15), 10–13.	England	6	Semi structured interviews	Novice nurses
Brekelmans, G., Poell, R. F., & van Wijk, K. (2013). Factors influencing continuing professional development: A Delphi study among nursing experts. European Journal of Training and Development,37 (3), 313–325.	Netherlands	38	Interviews	nurse managers, professional associations
Clark, E., Draper, J., & Rogers, J. (2015). Illuminating the process: enhancing the impact of continuing professional education on practice. Nurse Education Today, 35 (2), 388–394.	England	35 + 31	Interviews	Nurses, managers, educators
Cleary, M., Horsfall, J., O’Hara-Aarons, M., Jackson, D., & Hunt, G. E. (2011). The views of mental health nurses on continuing professional development. Journal of Clinical Nursing, 20 (23–24), 3561–3566.	Australia	50	Face-to-face interviews	Nurses
Draper, J., Clark, L., & Rogers, J. (2016). Managers’ role in maximising investment in continuing professional education. Nursing Management, 22 (9).	UK	35	2 step semi-structured telehpone interviews	Nurses, managers,
Ennis, G., Happell, B., & Reid-Searl, K. (2015). Enabling professional development in mental health nursing: the role of clinical leadership. Journal of psychiatric and mental health nursing, 22 (8), 616–622.	Australia	12	Individual face-to-face interviews	Mental Health Nurses
Fairchild, R. M., Everly, M., Bozarth, L., Bauer, R., Walters, L., Sample, M., & Anderson, L. (2013). A qualitative study of continuing education needs of rural nursing unit staff: The nurse administrator’s perspective. Nurse education today, 33 (4), 364–369.	USA	40	Qualitative interviews	Nurse administrators
Goudreau, J., Pepin, J., Larue, C., Dubois, S., Descôteaux, R., Lavoie, P., & Dumont, K. (2015). A competency-based approach to nurses’ continuing education for clinical reasoning and leadership through reflective practice in a care situation. Nurse education in practice, 15 (6), 572–578.	Canada	85	Descriptive longitudinal evaluative research design	Nurse managers, nurses and novice nurses
Govranos, M., & Newton, J. M. (2014). Exploring ward nurses’ perceptions of continuing education in clinical settings. Nurse Education Today, 34 (4), 655–660.	Australia	23	Case study	Clinical nursing staff
Gray, M., Rowe, J., & Barnes, M. (2014). Continuing professional development and changed re-registration requirements: Midwives’ reflections. Nurse education today, 34 (5), 860–865.	Australia	20	Longitudinal case study in depth qualitative interviews	Midwives
Green, J. K., & Huntington, A. D. (2017). Online professional development for digitally differentiated nurses: An action research perspective. Nurse education in practice, 22, 55–62.	New Zealand	10	Action research approach	Nurses
Shrestha, G. K., Bhandari, N., Singh, B. (2010). Nurses’ views on need for professional development in Nepal. Journal of the Nepal Medical Association, 49 (179).	Nepal	11	Focus group interviews	Nurses
Jantzen, D. (2019). Refining nursing practice through workplace learning: A grounded theory. Journal of clinical nursing, 28 (13–14), 2565–2576.	Canada	17	Semi structured interviews	Experienced nurses
Jho, M. Y., & Kang, Y. (2016). Perceptions of continuing nursing education in Korea. The Journal of Continuing Education in Nursing, 47 (12), 566–572.	Korea	17	Focus group interviews	Nurses
Kyrkjebø, D., Søvde, B. E., & Råholm, M. B. (2017). Nursing competence in the municipal health service: can professional development be accommodated?. Norwegian Journal of Clinical Nursing/Sykepleien Forskning.	Norway	14	Focus Group	Nurses
Lee, N. J. (2011). An evaluation of CPD learning and impact upon positive practice change. Nurse Education Today, 31 (4), 390–395.	UK	11	Interviews	Nurses
Pool, I., Poell, R., & ten Cate, O. (2013). Nurses’ and managers’ perceptions of continuing professional development for older and younger nurses: A focus group study. International journal of nursing studies, 50 (1), 34–43.	Netherlands	22	Focus group interviews	Nurses, managers
Pool, I. A., Poell, R. F., Berings, M. G., & ten Cate, O. (2015). Strategies for continuing professional development among younger, middle-aged, and older nurses: A biographical approach. International journal of nursing studies, 52 (5), 939–950.	Netherlands	21	Semi-structured interviews	nurses at different career stages
Pool, I. A., Poell, R. F., Berings, M. G., & ten Cate, O. (2016). Motives and activities for continuing professional development: An exploration of their relationships by integrating literature and interview data. Nurse education today, 38, 22–28.	Netherlands	21	Semi-structured interviews	nurses
Price, S., & Reichert, C. (2017). The importance of continuing professional development to career satisfaction and patient care: meeting the needs of novice to mid-to late-career nurses throughout their career span. Administrative Sciences, 7 (2), 17.	Canada	185	Focus group interviews	nurses at different career stages
Stanford. How can a competency framework for advanced practice support care? British Journal of Nursing, 2016, Vol 25, No 20	UK	8	Qualitative cross-sectional design	Nurses
Tame, S. L. (2011). Secret study: A new concept in continuing professional education. Nurse Education Today, 31 (5), 482–487.	UK	23	Interviews	Perioperative Nurses
Thurgate, C. (2018). Supporting those who work and learn: A phenomenological research study. Nurse education today, 61, 83–88.	UK	46	Interviews	Nurses

e) data analysis, which is explained in more detail below, and.

f) expressing the details of the synthesis which is done in the findings sections below.

### Search strategy

A comprehensive systematic search of literature was subsequently conducted on Medline (OVID), PubMed and Cumulative Index to Nursing and Allied Health Literature (CINAHL), Web of Science (Clarivate) and ERIC (ProQuest). The literature search was conducted by a librarian. The literature search was conducted in December 2019 and was limited to articles published in English from 2010 to 2019. Inclusion and exclusion criteria for the literature search were established and are presented below in Table 2. The inclusion criteria comprise of articles from empirical studies (using qualitative methods), discussing nurse continuing learning and education, professional development, lifelong learning, CPD, motivation and barriers.

### Data analysis

**Fig. 1:**
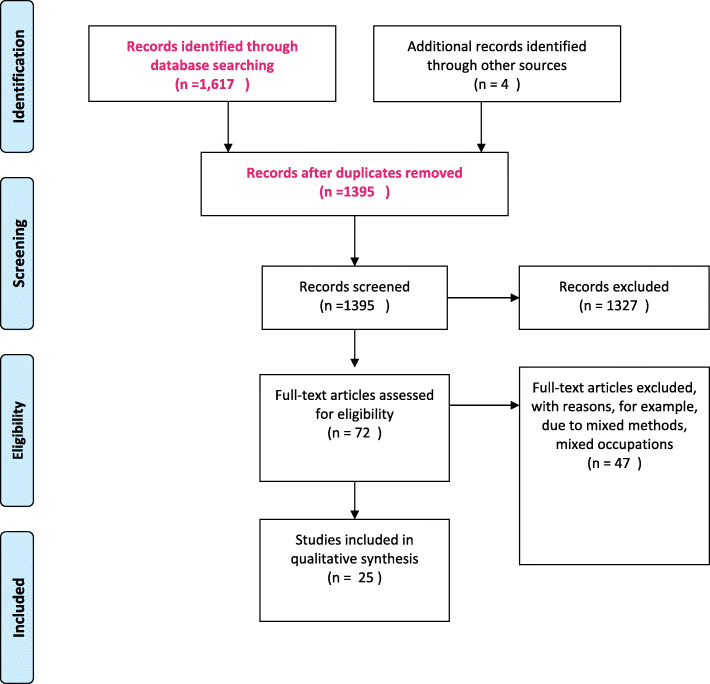
Overview of the steps in the literature screening

A total of 1675 records were identified, and following de-duplication, 1395 articles remained. All 1395 articles were screened. Articles had to address nurses’ CPD and continuing education, using qualitative oriented methods. After the first screening 72 articles remained. These articles were divided into three batches and were divided among the researchers. Each author read one batch to further identify if the articles were to be included. For each batch, a second author read the articles, meaning all articles were read by at least two authors. Any remaining ambiguities were discussed and resolved among the team. Figure 1 is a summary of the literature search and screening and Table 3 presents an overview of each study with its citation, location, cohort size and data collection method. 25 articles were identified for the final metasynthesis. All authors read the final 25 articles. Quality assessment using CASP criteria as outlined by Lachal et al., [36] is reported in Table 4. In the quality assessment we assess the following components; Was there a clear statement of the aims of the research?, Is a qualitative methodology appropriate?, Was the research design appropriate to address the aims of the research?, Was the recruitment strategy appropriate to the aims of the research?, Were the data collected in a way that addressed the research issue?, Has the relationship between researcher and participants been adequately considered and reported?, Have ethical issues been taken into consideration?, Is there a clear statement of findings? We also introduce the question of whether the texts are available in Open Access form or not. We introduce this question, as we believe the outcomes on research on nurses’ perceptions and experiences of CPD is potentially important for their practice, and access via Open Access channels could act as a quality dimension. However, without access to the data and the process of interpretation we choose not to assess; How valuable is the research?, Was the data analysis sufficiently rigorous?

**Table 4:** Quality assessment according to modified CASP criteria

Criteria	Totally met	Partially met	Not met
Was there a clear statement of the aims of the research?	18	3	
Is a qualitative methodology appropriate?	21		
Was the research design appropriate to address the aims of the research?	18	3	
Was the recruitment strategy appropriate to the aims of the research?	17	4	
Were the data collected in a way that addressed the research issue?	18	3	
Has the relationship between researcher and participants been adequately considered and reported	2	2	17
Have ethical issues been taken into consideration	9	10	2
Is there a clear statement of findings?	20	1	
Available Open Access	18		3

For the final analysis enabling the synthesis of the studies in this metasynthesis the articles were read carefully, findings related to the research questions; What is the reported value of CPD for nurses’ lifelong learning and its impact on nursing knowledge?, what are the conditions necessary for CPD? And what are the challenges faced by nurses when engaging in CPD?, were identified. In the next step of the analysis, study findings were examined using constant comparative analysis. The findings and conceptual categories were coded, compared, and sorted, focusing on conditions, strategies, and consequences. Finally, the synthesis, the interpretation of the findings, were described as themes, and these were revised several times until a coherent whole was formed [30, 36–38] Before the final description of the synthesized themes, all the three authors discussed the content of the themes until consensus concerning credibility was reached.

### Findings

From the metasynthesis we present five overarching themes, Organisational culture shapes the conditions, Supportive environment as a prerequisite, Attitudes and motivation reflect nurse’s professional values, Nurses’ perceptions of barriers and Perceived impact on practice as a core value. Each theme is further explained below with references to the relevant literature.

### Organisational culture shapes the conditions

Organisational culture played an important role towards the professional development of staff. Organisational commitment and support to personal and professional development of its staff was seen as an indication that staff were valued [5, 15] Moreover, CPD initiatives contributed to attracting and retaining staff [39]. Additionally, a culture that was flexible and adaptable to change was perceived by some participants to be favourable towards CPD [40–42]. Flexibility extends to matters such as CPD availability, and also location, but related also to creating opportunities in the work schedule for the nurses to participate [43]. Other organisational factors such as funding for CPD programs, staff access of CPD learning, role of management in staff CPD, manageable nursing workloads, the design & delivery of CPD activities, communication and collaboration between CPD providers and management are specifically organisational factors seen as crucial to effective staff development [44, 45]. Developing a strategy for CPD was also acknowledged as a key element of organisational culture as a way of enabling participation [46]. In a similar fashion, it was argued that the organisation needs to be focussed on incremental, but constant development of practices, and here CPD was seen to play a key role [47]. This sentiment was expressed elsewhere too, but from a re-skilling, or keeping up-to-date perspective, where the organisation is seen to have great importance [48, 49]. The value of partnerships and shared understanding between managers and nurses as key enabling factors was identified in several studies [46, 50]. In a related fashion, Jantzen argues that organisations should actively avoid fragmentation of CPD initiatives [51]. As more CPD training is digitised IT/ICT (information communication technology) skills were seen as key to successful CPD implementation [46, 52]. It was acknowledged that the transformation to online learning does not only affect nurses, it involves change for the whole department [52].

### Supportive environment as a prerequisite

An environment that supports learning was seen as a necessary prerequisite for CPD. Conditions had to include, flexible off-duty patterns to allow time for staff to study, availability of workplace learning, workloads were not excessive and CPD was fully funded or a shared responsibility between employer and staff [46, 52]. Other indicators of a supportive environment included staff access to different CPD activities relevant to their career goals, while at the same time meeting organisational goals and where staff felt free to study openly and not secretively [15, 41]. Moreover, the development of local and contextual CPD was seen as something that supported and made participation possible [43, 53, 54]. Participants indicated that nurses required financial support and practical support in the form of adequate time to participate in CPD activities and suitable staff cover when colleagues were away attending CPD activities [47]. Jantzen et al. [51] suggest there are three catalysts in a supportive environment; mentors, workplace camaraderie and a highly functional workplace team. Moral support or encouragement was identified in more than one study, where it was articulated that learners want to know there is an appreciation for the time and dedication needed to engage in CPD [44, 46, 50]. The value of learning from other health professionals other than nurses, in the day-to-day work was highlighted for professional development [54]. Similarly, the sense of a supportive environment with a strong team spirit is communicated elsewhere [39]. Explicit support is noted in several studies; support for novice nurses [39] but also the importance of explicit managerial support [55]. Conversely, in one study, respondents noted that there was less support for experienced or late career nurses [56].

### Attitudes and motivation reflect nurse’s professional values

The value and importance of CPD was discussed in many of the studies. In some, CPD was perceived to be key in defining nurse professionalism [6, 15, 40, 47, 49]. Engaging in CPD was also viewed by new nurse graduates as an important element of their individual professionalisation in nursing [6, 15, 40]. In addition, CPD was perceived to be important for enhancing and up or re-skilling, keeping knowledge and skills up-to-date, considering that nursing practice has become more evidence based [6, 43, 46, 51, 54, 56]. Furthermore, nurses stated that CPD was important for maintaining licensure, and felt that the responsibility for enrolling and participating in CPD activities was with the individual nurse, not with the employing organisations [53]. On the other hand, participants felt more motivated to learn if they could easily access CPD programs, if they felt supported and if there were a variety of CPD activities on offer. Here, bedside and informal learning was emphasized as important [57]. Similarly, contextualising learning and placing it in close proximity to practice was seen to enhance motivation and engagement [42]. CPD was also viewed as a way to start networking with other peers [44]. In one study, a competency framework was introduced, here participants felt that such a framework could help them reflect on their own practice and, as it provides a systematic approach to assessing a patient, look at their own strengths and weaknesses [58]. Such competency frameworks help to harness scarce training more effectively and encourage individuals to take more responsibility for their own development [58].

Participants’ attitudes towards CPD funding were mixed, with some stating that funding for CPD was the employer’s responsibility, while others felt that the individual practitioner was responsible or that the burden ought to be shared between the organisation and the nurse [5, 15, 40].

### Nurses’ perceptions of barriers

Poor staffing levels, heavy workloads, lack of funding, lack of study time and anti-intellectualism were some of the perceived barriers to CPD brought out by this review. Participants in the studies reviewed felt that a lack of organisational support, especially from their managers, was an indication that the organisation did not take professional development of its staff seriously [46]. Some respondents reasoned that an anti-academic culture and lack of relevant CPD programs was further indication of this [5, 15, 40]. Seeing a connection to patient care was identified as a strong driver and nurses identified that CPD initiatives would be filtered out unless there was such a clear connection to patient care [43, 51].

Additionally, some studies indicated that as role models, managers had to show interest in their own CPD, in order to motivate other nurses. In other words, the manager’s knowledge of CPD activities was reflected by their attitude towards work-based study, acceptance of staff who studied openly, the way the manager prioritised funding support and managed staff shift schedules to allow study release time [5, 39, 54, 56]. Fatigue was identified as a major barrier. For example in Jho et al. [53], in a context of mandated CPD, respondents felt tired due to the heavy nursing workload in conjunction with CPD. Lack of strategy, and financial initiatives in terms of money, or time off to study was also acknowledged as a barrier [5, 39, 54, 56]. Lack of transparent career trajectories were also acknowledged as an area of concern [44].

Other barriers, or de-motivating factors were identified; difficulties in attending CPD and keeping a life-work balance [48]. Barriers included: formal CPD courses away from the clinical areas were perceived to lack in authenticity [47, 49] and a mis-match in expectations and outputs, where nurses viewed themselves as agents of change, but where the organisation was unable to offer means to capitalise on this perception and desire to bring about change [50, 59]. As much as competency frameworks were viewed positively in offering a sense of direction, a divergent view was that they were limiting or created set boundaries that participants experienced as limited, for example, if used as prescriptive, hindering nurses to define their own learning needs [58]. Lack of IT competence was also perceived as a barrier [52] with more CPD being conducted online.

### Perceived impact on practice as a core value

The impact of CPD on nursing practice was perceived as important and valuable in different ways. The impact could be both direct and indirect depending on the organisational culture [41, 45]. This mixed perception could be due to the complex nature of health care organisations which can make knowledge sharing difficult [45] and that some CPD learning was done secretly, results of which were difficult to evaluate [41]. In the case where a competency framework was studied, participants felt that using the competency framework helped them organise their work and their thought processes [58]. A common sentiment was that CPD would benefit health care organisation through the provision and enhancement of practitioners’ knowledge and skills [46]. Sentiments articulating expectations of an impact of CPD could also be seen elsewhere too [52, 55, 56, 60]. Moreover, CPD is expected to rely on better communication between managers and nurses as a way of informing each other about needs and means of fulfilling those needs [48]. Direct impact was realised through improved interprofessional collaboration and the idea that new methods could be directly translated into practice [47]. Others however, raised concerns that CPD programmes or courses may not translate into new practices [50]. This sentiment was echoed elsewhere too, where a need to situate CPD in close proximity of patients was seen as important for CPD to impact practice [49] While indirect impact happened through dissemination of knowledge and skills from CPD learning to other nurses at ward level, arguments were put forward that there will be no difference to practice unless organisational processes support and evaluate its effect on practice [46]. Participants reported that their professional confidence was enhanced, they felt they could challenge medical decisions and the status quo [41]. Furthermore, participants felt that CPD enhanced their professional knowledge and skills for better patient care through improved care standards, how they communicated and collaborated with other professionals. Participants also believed that learning increased their chances for career progression and reduced work-related anxiety because of enhanced knowledge [40, 41].

## Discussion

The aim of this paper is to conduct a metasynthesis investigating the qualitative research on nurses’ experiences of continued professional development. As a result, this metasynthesis revealed a number of overarching themes, which synthesize the findings of previous qualitative oriented research during the period 2010–2019. 2010 was chosen to include the last 10 years of CPD research. The themes are; Organisational culture shapes the conditions, Supportive environment as a prerequisite, Attitudes and motivation reflect nurse’s professional values, Nurses’ perceptions of barriers and Perceived impact on practice as a core value. The themes put focus on important issues that were recurrently put forward by the nurses in the studies reviewed. However, the themes are not isolated from each other, rather, the content of the themes is interrelated. Some of the themes mainly mirror an overarching perspective at the organisational level of health care, while other themes describe the nurses’ experiences and needs on a personal level. The following discussion explores the above themes in relation to the three questions posed earlier; what is the reported value of CPD for nurses’ lifelong learning and its impact on nursing knowledge? What are the conditions necessary for CPD? What are the challenges faced by nurses when engaging in CPD? While we acknowledge that the questions and themes overlap, we have endeavoured to frame the discussion around the three research questions individually.

### What is the reported value of CPD for nurses’ lifelong learning and its impact on nursing knowledge?

Nurses reported that CPD raises professional standards through competencies gained, thereby increasing professional performance with positive benefits for patients, organisations and individual nurses [40]. These outcomes were seen most prominently in the themes Attitudes and motivation reflect nurse’s professional values, and Perceived impact on practice as a core value. Closely aligned to CPD are the nurses’ clinical effectiveness and competence. Maintaining both requires nurses to keep their practice up-to-date highlighting the importance of CPD for nurses. The knowledge and skills gained by nurses through CPD advances the professional status of nursing, which was an idea that was prevalent in some of the studies in this review [15, 40, 47, 50], but is also illustrated elsewhere in the literature [8, 21]. Nurses acknowledged that expectations of professional accountability meant that standards of practice ought to be kept high in order to pass public scrutiny [15, 40]. Furthermore, skills acquired through CPD, such as the ability to conduct systematic peer-reviews [45] and appraise literature for best evidence, provide nurses with essential professional competencies, embeds values such as caring behaviours, influences beliefs and attitudes which in turn shape nurses’ professional conduct [61]. As such CPD is seen as a tool for nurses to update their skills, and in doing so deliver safe and high-quality health care. As revealed in this review, nurses were willing to fully fund or part-fund their CPD as long as CPD programs were captivating, easily accessible, there was fair allocation of study time and their efforts towards CPD were recognised. The latter implies that nurses want time and space to transfer their CPD learning into practice and for their CPD to be recorded [5, 45]. The belief is that, consequently, patient care will improve with positive impact from organisational change [15, 45]. However, it is clear that the organisation is key in making CPD work for nurses. The issues brought up in the theme organisational culture shapes the conditions is thus very important in stimulating nurses to engage in CPD. The nurses’ attitudes and motivation to engage in CPD also depends on a supportive environment and engagement may in turn influence the organisational culture.

### What are the conditions necessary for CPD?

A disconnect could be seen in relation to the conditions for CPD, where access to CPD training came to the fore as problematic in some of the studies. Nurses had to travel long distances to attend courses [15, 62, 63]. To avoid these challenges, nurses settle for CPD as long as it fulfils mandatory requirements for registration [53]. If intentions of CPD are to provide a basis for the continual updating of skills, then authentic learning as an expected outcome is seen as a prerequisite for nurses to engage in CPD, whether it occurs at the bedside, at a training facility or through an IT mediated interaction. This calls for accessible CPD, improved design and delivery methods for all nurses [52]. Nurses’ experiences described in the themes Organisational culture shapes the conditions, Supportive environment as a prerequisite, show that structural and moral support are both important. Structural support in the form of availability, time to engage in CPD, as well as clear expected outcomes [46, 49], but also moral support in the form of an understanding management and environment, and also peers and leaders who themselves also prioritise CPD [58]. Organisational support and commitment towards CPD should mean allocation of study time, support of nurses who study privately, by creating space for knowledge and skills integration and managing poor cultural practices that hinder open study. Funding is seen as a key factor across many of the studies, both in terms of enabling nurses to participate, but also as a way of acknowledging nurses who engage in CPD. Further studies may need to look more closely at how nurses perceive different aspects of funding. For nurses’ lifelong learning to endure, CPD programs need to be more accessible and kept interesting by making them more relevant to nurses’ practice contexts. Here the importance of the organisation for creating a CPD conducive environment is emphasized [46, 51, 52]. As role models, managers need to lead by example and engage in CPD themselves, but also demonstrate explicit support. They also need to influence policy to create environments conducive to CPD. If funding situations do not improve, work-based CPD learning could be one of the alternative ways of CPD delivery for nurses. To promote CPD engagement and cost reduction, eLearning approaches could be utilised for education and training. However, poor IT skills among nurses, but also within organisations continues to be a potential weakness [52]. A challenge remains here in enabling nurses to get recognition from informal on-site learning [16–18], where elements of meta-cognitive reflection can be used to acknowledge nurses’ continued professional development.

### What are the challenges faced by nurses when engaging in CPD?

In some of the literature reviewed, participants lamented their current conditions for CPD, and identified clear barriers and challenges in the form of concerns related to lack of funding for CPD, staffing levels, time allocation for study, lack of organisational support because of negative cultural practices, CPD design & delivery and limited choice of CPD activities. This is articulated within the themes: Organisational culture shapes the conditions, Supportive environment as a prerequisite, Nurses’ perceptions of barriers [2, 11, 34, 41] . However, studies did not explore the views of nurses on recruitment and retention and its impact on accessing a variety of CPD activities. Evidence from this review indicates that modernising healthcare and simultaneously cutting CPD funding for nurses could lead to a limited number of nurses attaining the skills and competences needed for the modernisation process. In view of the understaffing that is reported elsewhere [5, 15], we identify a cause for concern. These perceived barriers may undermine nurses’ professional development [23, 59]. Moreover, the findings presented here revealed that nurses face a number of challenges in relation to their CPD participation. The challenges include limited CPD activities to choose from, poor CPD delivery methods, negative organisational culture practices such as anti-intellectualism and lack of support. As a result, nurses were less motivated to participate in CPD training [57].

It is clear from the review, that IT concerns are becoming more and more prominent, given that more CPD programmes are being offered through digital platforms [47]. This is a concern for both the individual nurses, but also their organisations. On concerns regarding CPD delivery methods, nurses indicated that they preferred different styles. With these concerns comes the view that learners learn in different ways depending on the context and subject of study [61, 62]. This supports the notion that individuals have different learning preferences [61], where some adult learners learn better in a structured and teacher guided context, while others prefer self-direction.

### Limitations

The search was conducted by an experienced search engine expert. Even so, we may still have been unsuccessful in finding all the relevant articles. The study was focussed on qualitative studies, which means that studies using predominantly quantitative or mixed methods were not included, but could hold important insights. In the introduction to the study we used the UK as an example for how CPD might be regulated. However, we have conducted a comprehensive search of the literature and our analysis was not conducted with a UK-centric perspective. While each study needs to be understood in terms of local rules and regulations, the similarities in the findings are striking.

## Conclusion

The metasynthesis indicates that differences exist between the nurses’ CPD needs and expectations and organisations’ approaches to nurses’ professional development. The review lays bare a disconnect between the rhetoric of identifying CPD as a way to enhance nurses’ skills, and the reality of CPD interventions, where nurses do not feel support within their organisations or from their immediate supervisors. The review also revealed that CPD is an important element of nursing practice and nurses’ lifelong learning. Furthermore, it suggests that nurses are motivated to take part in CPD to enhance their knowledge, improve skills and keep up- to -date with recent evidence. While evidence from this review indicates that nurses believe that CPD has a positive impact on patient care, there is lack of contemporary research to qualify this claim and there is limited evidence from this review to support this assumption. However, evidence from the review suggests and confirms, that the greatest barriers for CPD in nursing are a lack of funding and time to participate in CPD activities, which are clearly related to organisation structure. It is difficult to envisage how such conditions could be conducive for nurse CPD to flourish. Such perceived barriers undermine nurses’ efforts to keep knowledge and skills up-to-date and provide better patient care while meeting the ever-changing needs and expectations of their patients. This is further exacerbated by negative organisational cultural practices and lack of knowledge on how to facilitate, design and deliver CPD for their staff. We conclude that policy makers and relevant stakeholders need to put in place strategies to support nurse CPD in long term and in doing so tear down the barriers of CPD.

## Data Availability

The data in the study is comprised of previous research articles. A full list of articles is included in the Table 3.

## Abbreviations

CPD: Continued Professional Development
UKCC: United Kingdom Central Council for Nursing, Midwifery and Health Visiting
0PREP: Post registration education and practice
NMC: Nursing & Midwifery Council
HEE: Health Education England
NHS: National Health Service
